# A qualitative exploration of people living with dementia's experiences of using everyday technology

**DOI:** 10.1177/20552076251351538

**Published:** 2025-06-30

**Authors:** Annabel Ditton, Shirley Evans, Christopher Fox, Jane Cross

**Affiliations:** 1 3286 Faculty of Health and Life Sciences, University of Exeter, Exeter, UK; 2 8709 School of Health and Wellbeing, Worcester University, Worcester, UK; 3 6106 School of Health Sciences, University of East Anglia, Norwich, UK

**Keywords:** Dementia, technology, qualitative, digital, tailoring, electronic

## Abstract

**Background:**

Technology is becoming a popular and cost-effective way of supporting people living with dementia. Despite this, uptake and adherence to technology interventions is variable. Two factors contributing to this are people's pre-existing attitudes towards technology which influence how accepted interventions are, and how accessible technologies are to use. Attitudes and accessibility are developed through people's everyday experiences of technology which are underexplored in research.

**Method:**

This study used photo-elicitation interviews to explore ten people living with dementia's experiences of using everyday technology and dementia-specific technology. Experiences were explored in and outside the home environment to understand experience at an individual and societal level. Reflexive thematic analysis was used to understand how technologies are experienced, and how these experiences might contribute to the acceptability and accessibility of technologies supporting people living with dementia.

**Findings:**

Four overarching findings were formed from the data: 1) Impact of symptoms of dementia on technology use, 2) Motivation to use technology, 3) The importance of integrating appropriate technology, and 4) The importance of setting in technology implementation.

**Conclusions:**

These findings demonstrate that technologies are a desirable method of providing support to many people living with dementia if the correct tailoring and support is given. People living with dementia are motivated to use technology for various reasons, but interventions that enhance existing face-to-face services, rather than replace them maybe more accepted. Finally, people living with dementia are hopeful that technology will help future generations because these individuals will begin their dementia journey already equipped with the confidence and skills to manage more complex technologies effectively.

## Introduction

Maintaining and improving the quality of life of people living with dementia remains a national priority.^1,2^ Technology as a means of providing support could be a cost-effective solution for service providers.^3–5^ So far, developments in assistive technologies, cognitive training, reminiscence applications, and exergames have demonstrated promising outcomes in relation to cognition, mood, physical activity, and social relationships.^6–10^ However, uptake of interventions is variable with numerous barriers leading to lack of adherence to recommendations.^11,12^ Barriers include the personal cost of and lack of appropriate equipment,^13,14^ poor digital literacy or anxiety,^11^ and cognitive impairments.^15^ A factor acknowledged but less explored are pre-existing attitudes towards technology held by people living with dementia.^16^ Older adult and dementia carer literature suggests positive attitudes lead to willingness to adopt technology.^17,18^ Social psychology theory suggests attitudes are formed by combinations of affective (e.g. values, feelings and beliefs), cognitive (e.g. social environment and experience), and behavioural concepts.^19,20^ This suggests that positive everyday technology experiences might make technologies for supporting people living with dementia more desirable.

If true, exploring people living with dementia's experiences of everyday technology is important to understand how and why technology interventions are, or are not, accepted by people living with dementia. ‘Everyday technologies’ is a term used to describe the variety of technical, electronic, and mechanical equipment that exists in our everyday lives.^21^ Most studies have explored experiences of using specific dementia technology interventions, rather than general everyday technology experiences.^22–25^ However, research indicates that everyday technologies are more commonly used than specialised dementia technologies.^26^ People living with dementia's experiences of some everyday technologies were explored by Liddle and colleagues;^27^ however, these focused on technologies to support communication and participation, which may not account for all types of everyday technology or all the reasons people living with dementia use technology.^26^ This study qualitatively explores people living with dementia's experiences of using a wide variety of everyday technologies and technologies designed to support dementia using photo-elicitation. Using photo-elicitation to explore everyday technology experiences of people living with dementia is novel, and focus was placed on exploring the affective, cognitive, and behavioural concepts that contribute to technology experiences.

### Study objectives

- To explore how people living with dementia experience their interaction with technology in their daily lives.
- To explore how people living with dementia understand technology and its role in wider society.

## Methods

### Design

This work was a photo-elicitation study using social constructionism. Influenced by empowerment theory the research design focused on promoting wellness, competence, and strengths.^28^ We used photo-elicitation interviews to collect participants technology experiences which involves introducing a photograph into the research interview.^29^ This was chosen because it promotes flexibility and creativity, favourable to compensate for traditional methods that can exclude those with disabilities.^30–32^ Photographs taken by participants were used to represent their technology experiences and data were analysed using reflexive thematic analysis (RTA)^33^ acknowledging theoretical, social, historical, and cultural influences on knowledge construction and highlighting the role of the researcher within this construction.

#### Patient public involvement

Three people living with dementia and a group of twelve older adults and carers were consulted in the development of this study. These individuals consulted on the feasibility of using photo-elicitation, improved the accessibility of recruitment resources, and ensured that written camera training was understandable. Additionally, one individual living with dementia iteratively piloted and refined the interview topic guide to ensure opening questions were clear and did not rely on accurate recall. They also supported the analysis by refining and naming themes to avoid jargonistic terms.

### Participants

Based on previous examples of photo-elicitation and RTA, a sample size of 11 people living with dementia was expected to provide data sufficient for researchers to fulfil the research aims.^31,34,35^ Sufficiency, which refers to data richness and analytical rigour, was used because it acknowledges the socially constructed nature of experience making saturation impossible, and because data saturation is not compatible with RTA.^36,37^ Sufficiency was determined using the notion of information power^38^ and data quality was iteratively collected and analysed until we felt no new knowledge added to our understanding of the themes generated.

Participants were recruited using the Join Dementia Research database (JDR), the ‘dementia experts for involvement network for younger people living with dementia’, dementia community groups, and social media advertising. The study was advertised within these networks via email, posts in online forums, and face-to-face discussions. Participants were eligible if they were living in the community, they, or a family member self-reported a diagnosis of any type of dementia, and their dementia was mild to moderate. Living in the community was defined as living in their own home or somebody else's home with, or without support including those in sheltered housing or assisted accommodation, but not those in hospitals, nursing, or residential facilities. Dementia severity was determined through discussions between the participant and the researcher based on the descriptions of mild and moderate dementia taken from the JDR database (see supplementary material). Carers or supporters were not necessary for participants to take part;^39^ however, participants needed to demonstrate capacity to consent based on the Mental Capacity Act's guidance.^40,41^

Eleven Participants were recruited using convenience sampling; however, one withdrew following the briefing interview due to ill health. Researcher AD felt her interpretations of the data were rich enough to sufficiently fulfil the research aims at this point and thus made a situated and interpretative judgement to cease further interviews.^37,38^

### Materials

In photo-elicitation, images are part of the research process.^31^ Basic battery-operated cameras were offered to participants to avoid recruiting only participants owning cameras. Verbal, written and practical camera training was available; however, only two participants requested the cameras offered, and one used the camera to take their images. Others declined training opting to use their own camera or smart device to take pictures.

The interview schedule was indicative, and questions were free flowing and modified within and between each interview to create a conversational, friendly, and relaxed setting. This was important for generating high-quality data,^38^ and because such settings are characteristic of creating empowering research settings.^42^ Scheduled questions were therefore broad and follow-up questions were used to explore experiences in more depth as conversations developed.

### Procedure

This study was conducted across England throughout January 2024 to March 2024. Ten people living with dementia met with the researcher AD for what is described in photo-elicitation as a ‘briefing interview’.^31^ This lasted a maximum of 60 min and built rapport with participants, collected demographic information, checked eligibility, and provided informed consent. Participants were able to ask questions, problem solve potential challenges, discuss concerns, and receive camera training.

Participants were asked to take a maximum of 10 photographs portraying experiences of using everyday technology. They were encouraged to explore their technology experiences in and outside the home capturing both technologies they like and dislike and to be as creative as they wished. Participants returned between 8 and 10 images between 3 days and 2 months after the briefing interview. One participant requested to take part but taking photographs was too challenging, instead they were posted questions about technology experience and their responses used as visual prompts within the interview.

Participants completed a final interview using their photographs as prompts to name technologies and to describe what was in the photograph. These kept conversations focused, provided contextual references for participants when conversation trails were lost, and were an anchor for discussing broader abstract implications of technology. Seven participants participated using Zoom^©^, two in their homes, and one in a meeting room. Each interview lasted 90 min, with breaks offered regularly. Due to fatigue, one participant requested two shorter interviews each lasting 60 min.

### Ethical considerations

Ethical approval was provided by the University of Exeter's Research Ethics Committee (2871419). Informed consent was required from all participants with capacity determined at each interview in accordance with the mental capacity act.^40^ Capacity to consent was checked immediately prior to each interview. To demonstrate capacity, once going through the information sheet with the researcher (AD), participants had to summarise their required involvement in the study, weigh up the personal risks of taking part, and recall how their data will be used and stored.

### Data analysis

Audio recordings were used to transcribe interviews verbatim and analysed primarily inductively, meaning findings were created without pre-determined theory or frameworks guiding coding. To promote deeper reflections on the affective, cognitive, and behavioural concepts of experiences theme decisions were influenced by the theoretical lens of empowerment, Kitwood's^43^ theory of personhood, and by the researcher's previous experiences, knowledge, and social context. Kitwood^43^ highlights comfort, attachment, identity, occupation, and inclusion as key areas in which personhood can be maintained. Technologies which acknowledge individuality and balance personhood with care needs provide more empowering support.^44,45^ Key to this is adapting technologies that people already use to support people with dementia.^44^ Therefore, Kitwood's theory of personhood^43^ was used to guide analysis to produce findings which may better aid the future development of person-centered dementia technology.

As per RTA guidance^33^ researchers focused on identifying shared and contrasting thoughts, feelings and opinions towards technology and identified commonly used technologies and motivations for technology. This was completed using the six steps of RTA^33^:

Familiarisation was achieved by transcribing interviews, re-listening to audio clips, reading reflexive notes, and annotating initial thoughts, feelings, and ideas. To promote rigour during this step, researcher AD's reflections and observations were also combined with data using the principles of poetic transcription.^46,47^ Poems created were used to immerse her with data and reflect on her influence, not as a method of presenting data.

Coding was conducted by the researcher AD facilitated by NViVO (version 14). Initial codes were descriptive, portraying surface meanings and conceptual or implicit meanings^33^ after which patterns between codes were identified and codes grouped into similar or contrasting potential themes. Themes were reviewed by iteratively comparing them in relation to the coded extracts, and the full dataset.^33^ Refinement was supported by reflexive discussions with all authors and comparisons with existing theory. Themes and subthemes were then grouped into overarching themes. These were refined and named following iterative discussions amongst researchers to ensure themes were relevant and coherent. Reflexive discussions and an audit of decisions improved analytical rigour and trustworthiness.

To promote rigorous reporting, findings are reported using the COnsolidated criteria for REporting Qualitative research (COREQ)^48^ which can be found in the supplementary material.

## Results

This study explored daily technology experiences of 10 people living with dementia. Within the 10 participants (see table 1), there was an even split of males and females, and all resided in the UK. The mean age was 68 years, and 60% described their dementia as early onset. Participants had been living with dementia for between 2 and 10 years.

**Table 1.** Participant demographics.

Participant ID	Age	Gender	Ethnicity	Type of dementia	Years living with dementia
1	69	M	White British	Early onset Alzheimer's	5 Years
2	60	M	White British	Early onset Alzheimer's	5 Years
3	50	M	White British	Early onset Alzheimer's	4 Years
4	76	F	White British	Alzheimer's Disease	3 Years
5	51	F	White British	Primary progressive aphasia	4 Years
6	64	F	White British	Vascular Dementia	9 Years
8	70	F	White British	Early onset Alzheimer's	10 Years
9	68	M	White British	Mixed Dementia	9 Years
10	76	F	White British	Alzheimer's Disease	2 Years
11	74	M	White British	Alzheimer's Disease	2 Years

No participants had existing relationships with researcher AD and were unaware of her reasons for conducting the project prior to participation. Seven participants initiated contact with the researcher AD to express interest following advertisements and showed a clear knowledge of academia networks, were well established in dementia networks, and described interests in social change and dementia advocacy. The remaining participants were recruited from JDR. They chose to have a spousal partner present and requested face-to-face visits rather than videocalls. This group had been less involved in research and less exposed to dementia networks or advocacy.

Four findings were developed: (a) impact of dementia symptoms on technology use, (b) motivation to use technology, (c) the importance of integrating appropriate technology, and (d) the importance of Setting in technology implementation. These findings are explored in detail below and a summary is presented in table 2 to highlight key considerations for supporting people living with dementia to have successful experiences of everyday technology.

**Table 2.** Recommendations for supporting successful everyday technology experiences.

Theme	Considerations	Individual recommendation or influencer
Impact of dementia symptoms on technology use	Aging and dementia symptoms	Visual difficulties can make use challenging Cognitive impairments can make use challenging Implement and learn how to use technology early
	Applying strategies	Purchasing and tailoring devices to meet personal needs Make devices voice controlled Combine technology and non-technology solutions to meet personal needs
	Support	Family and friends People with technology knowledge Service providers
Motivation to use technology	Purpose	Technology fulfils a purpose such as maintaining personhood (Kitwood, 1997)
	Driven by social network	Family and friends advise or introduce new technologies Family or friends gift devices Service providers update or implement devices
	Previous experiences and skills	Experiences of technology in childhood including parents’ beliefs and feelings Extent of technology use during previous or current work/occupation Level of confidence in technology skills
Importance of integrating appropriate devices	Appropriate devices	Design of hardware is important for usability (e.g. Small, black devices may be more challenging) Simple software designs may be easier to use Devices fulfilling multiple functions are preferred by those with skills to use them Software continuity may make it easier to use devices Multiple security measures may be more challenging Devices must be affordable but still good quality
	Incorporating into lifestyles	Devices can be wired into the home Devices can have a physical ‘home’ within the environment to promote convenient access and regular use Linking devices together is useful for allowing convenient access and regular use Incorporating devices into routines is important to maintain skills
	Benefits of successful incorporation	Successful use may help avoid experiencing undesirable feelings and emotion (e.g. Anxiety, feeling like a burden) Multiple successes may build confidence in skills
The importance of setting in technology implementation	Negative beliefs about technology in society	People may have profound concerns about the impact of dementia on society People may believe technology negatively impacts human/face-to-face services Negative beliefs towards technology in society can lead to avoidance of technology
	Negative feelings towards technology in society	People may experience paranoia and fear about technology in society (e.g. Artificial intelligence, scammers) Negative feelings towards technology in society can lead to avoidance of technology
	Hopefulness about the future	Innovating current technology practices may improve how technology supports people living with dementia Technology may be a useful method of helping people living with dementia in the future

### Theme 1: Impact of dementia symptoms on technology use

This theme describes how challenges associated with normal aging, such as visual impairments, and symptoms of dementia make using technology more challenging because participants are unable to see aspects of the technology of forget how to use it. For example, a participant describes finding a computer keyboard challenging due to visual difficulties:

‘I sometimes struggle with seeing the, the letters and the numbers and the keyboard, but that's just my vision impacting’ (PWD_002).

**Figure 1.**
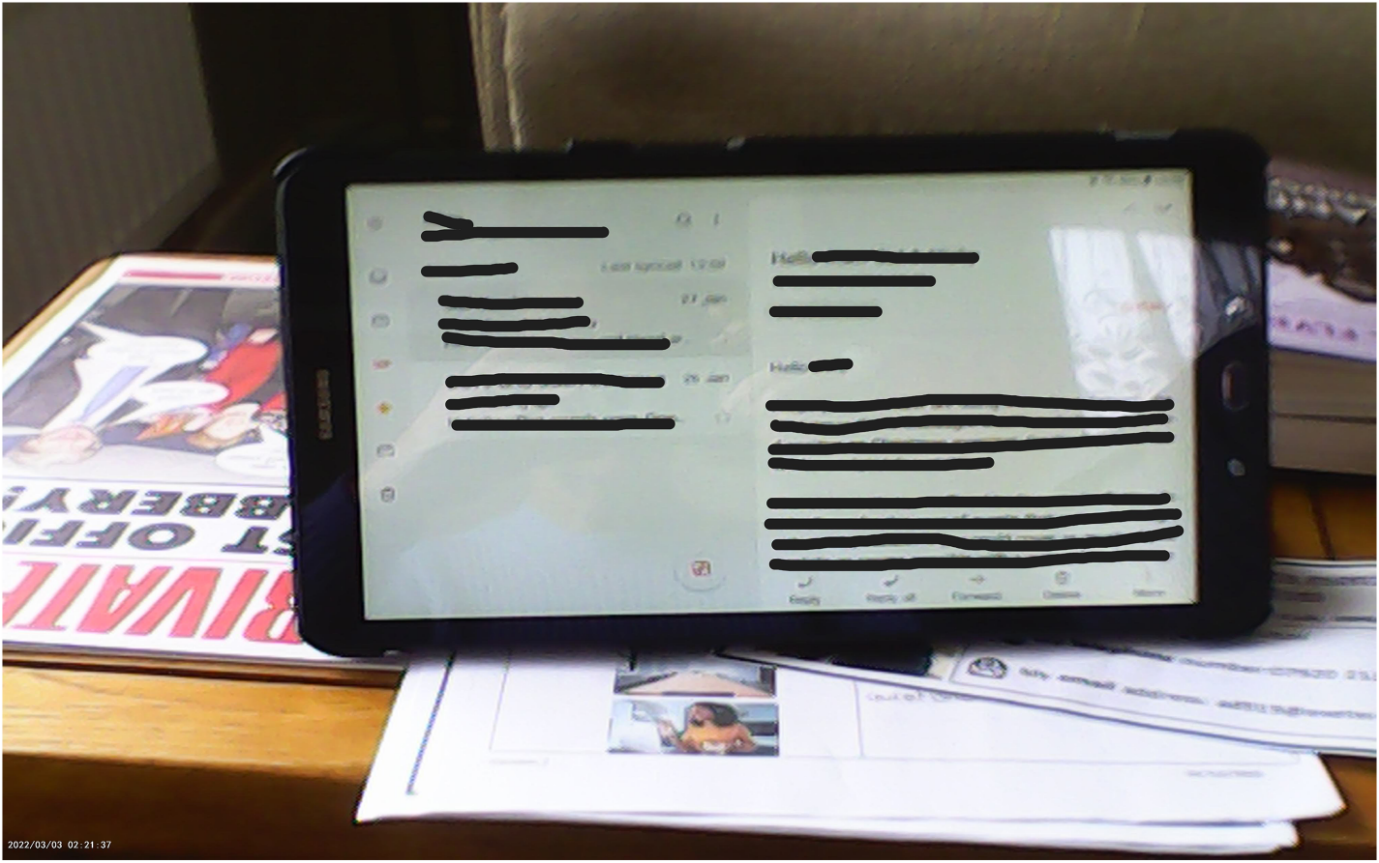
A participant's electronic tablet.

Similarly, another describes finding her tablet difficult because she forgets what she is doing (see Figure 1)*:*

‘it's difficult because I can, I'll, I'll be on the tablet and all of a sudden I'm thinking, what am I doing? You know, I, I can't. Up here [points to head] it's, it's telling me different things you see. That (…) it is annoying” (PWD_004).

**Figure 2.**
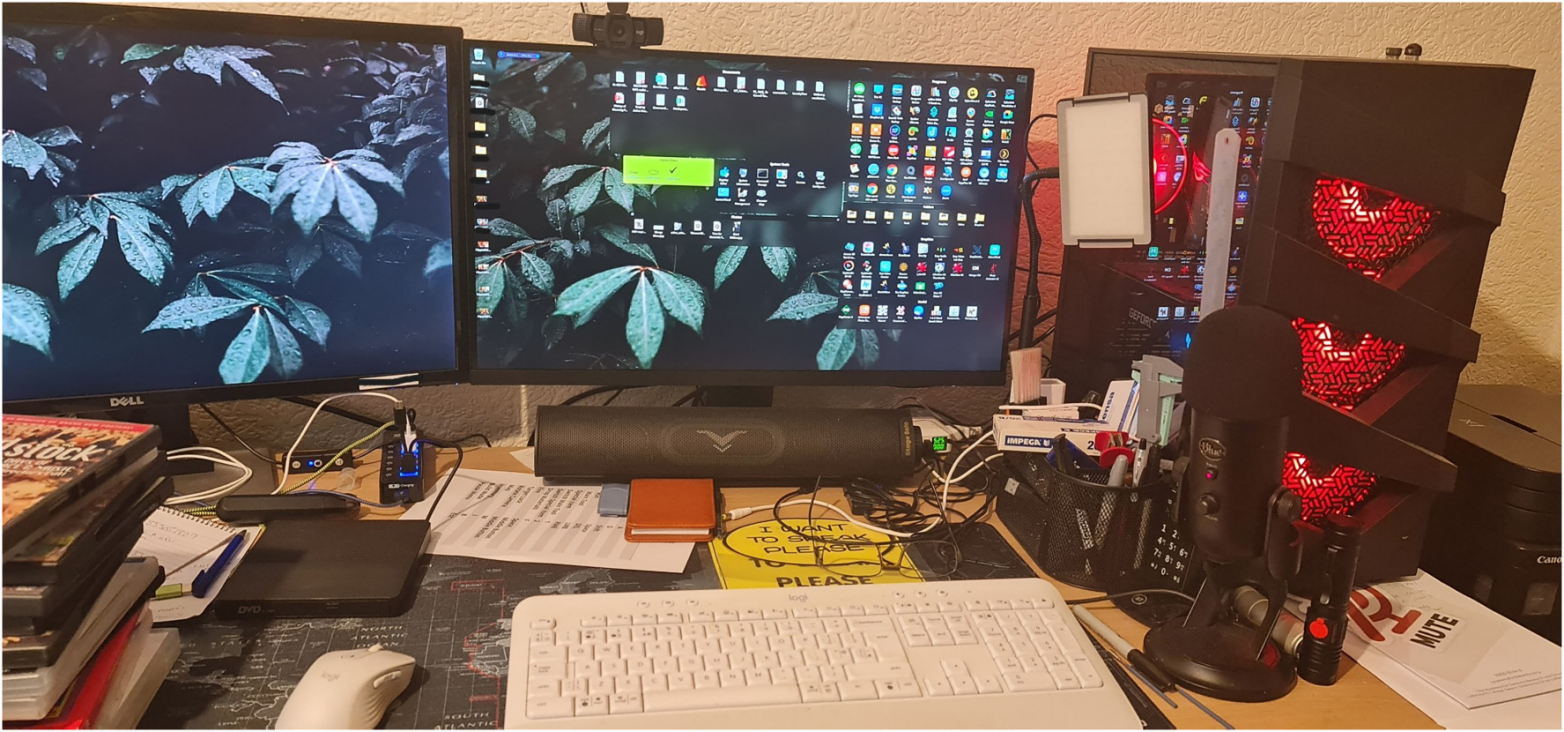
A participant's computer system which has been dynamically changed to suit their needs.

However, if motivated, individuals employ several strategies to preserve and maximise their ability to use technology for as long as possible*.* Firstly, participants adding to their devices to improve accessibility such as purchasing larger screens, or coloured keyboards to help with vision (see Figure 2):

‘I got it for, it's not as a gaming keyboard. It's a backlit keyboard and it has I think about 10 different colour settings and brightnesses. So you can have it whatever colour fits your, your visual needs at that time’ (PWD_009)

**Figure 3.**
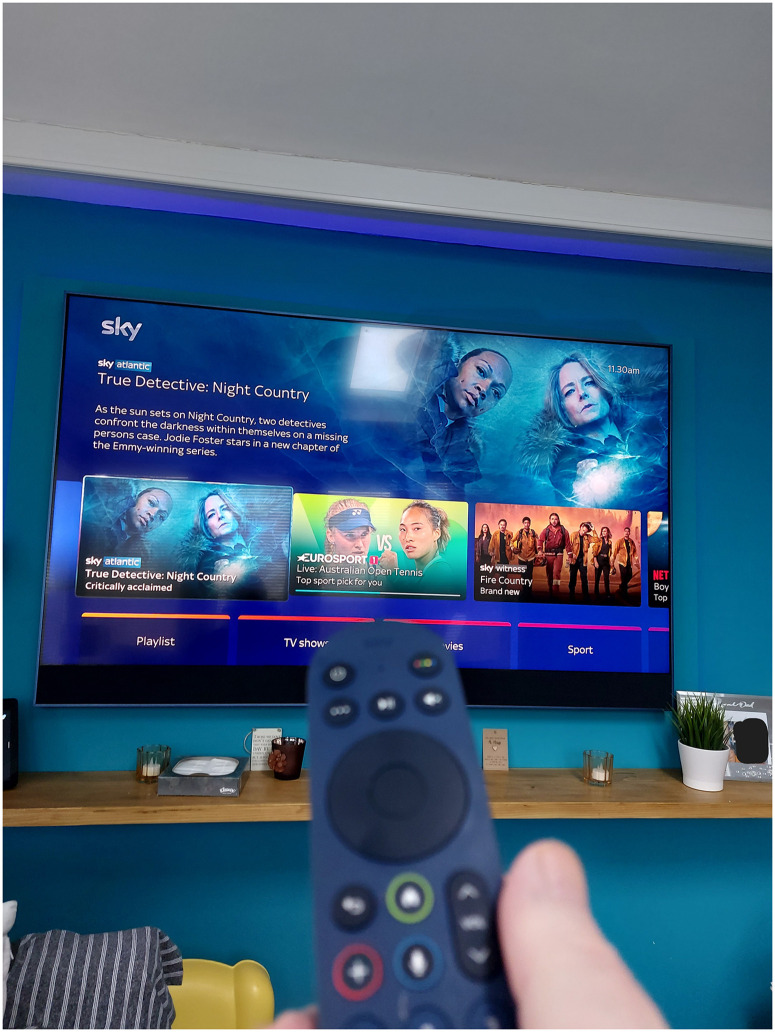
A sky TV remote that can be voice controlled.

Other strategies include buying or making devices voice controlled (see Figure 3):

‘It's getting bit more difficult now as, as the Alzheimer's progresses like, so I tend to more use verbal instructions now rather than the, rather the app on the phone because it's much more easier which is what, which is what it was intended for in the first place anyway’ (PWD_002).

These purchases and adaptations demonstrate important behavioural responses by participants to maintain accessibility meaning people benefit from technology for longer. Alternatively, people combine technology and non-technology solutions to find methods that work or them. For example, one participant uses his phone as a memory aid but prefers to physically go shopping because he enjoys keeping active:

‘Loads of photos because when you're shopping, making sure take a picture of the thing and I mustn't forget to buy’ … ‘I just prefer it because it's just nice to go out for quite a long walk around that shop for nearly an hour of exercise’ (PWD_011).

**Figure 4.**
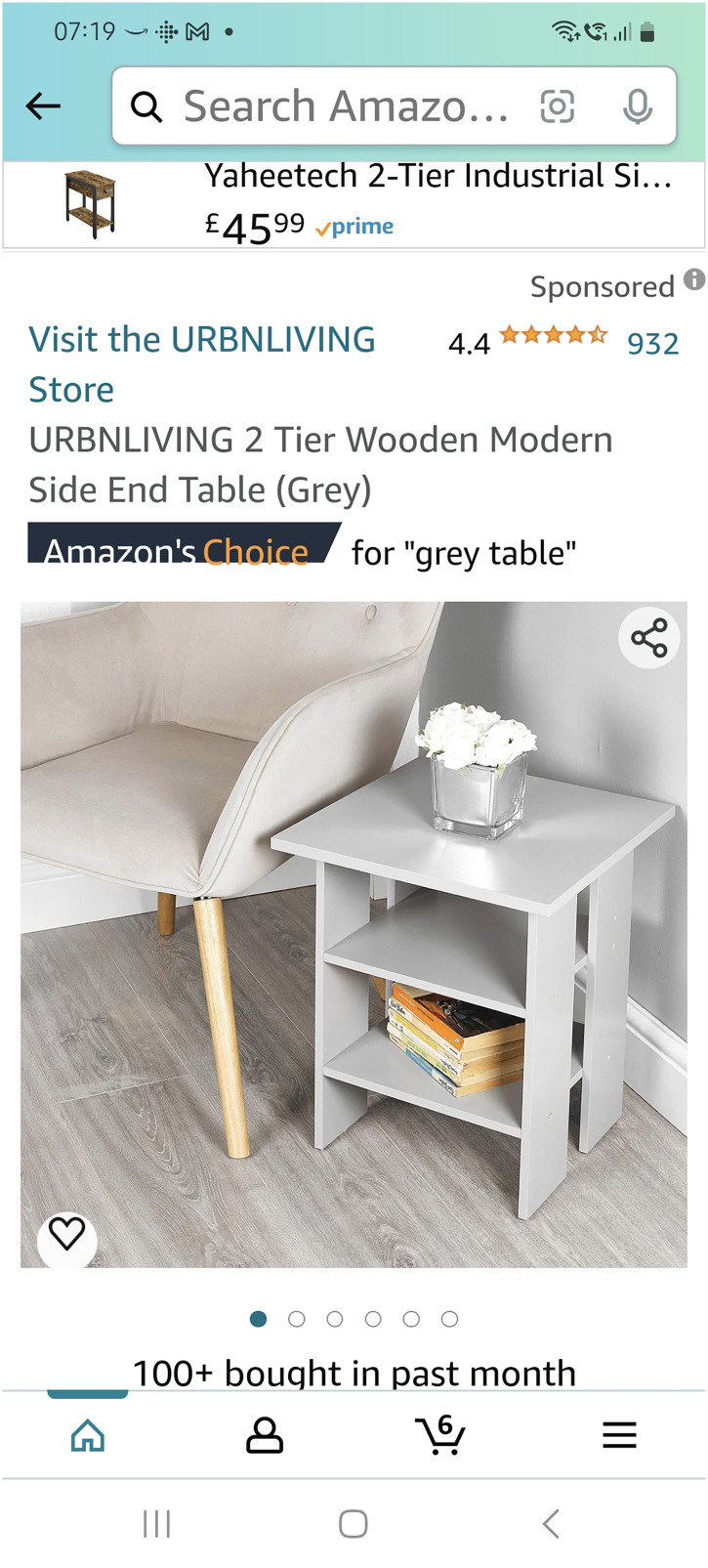
A screenshot of Amazon online shopping.

In contrast online shopping is preferred by some because it is convenient, despite recognising physically going to the shop being more cognitively healthy (see Figure 4):

‘It's easier than going to the shop now. From the dementia point of view going to the shops is actually more cognitively healthy because you're having to plan, you're going out, you're socialising, you're mixing with other people, … but from the, if you take that out and just say from the shopping point of view, no online is better’ (PWD_009)

These cognitive processes and behavioural combinations demonstrate how everyday technology use is tailored by people living with dementia to assist them. Therefore, it is important to understand individual's lifestyle priorities when implementing technologies.

Finally, when people living with dementia are unable to use technology independently, rather than give up, they seek support. Family members may be chosen to support for convenience and may be trusted. This means they tend to support with more intimate technology tasks such as banking or purchasing items:

‘I would write it down and that sort of thing. Would that benefit me? But I would speak to somebody and go look I want this because du-du-du. It's usually my son-in-law, I'd say, or my daughter would say I think this this gadget would really help me’ (PWD_008)

This also means they are most likely to be called upon if technology breaks because they are close by:

‘Actually, I'm quite lucky that my family are quite good on technology, aren't they? And if I got a little bit of a problem, I'll have a chat with them and then they'll just help me solve it’ (PWD_011).

People with technology knowledge are also chosen to support including charity workers, friends, neighbours, and technology providers. These individuals are seen as having adequate skills or used because support is offered as part of the company's policy. Support from these individuals is used to monitor device functioning and provide advice or practical support:

‘Ability net is a charity and it helps people with technology. Any age, any gender, any technology. Could be a phone, could be a telly, could be a dishwasher. So I rang up and they sent this [charity worker name]’ (PWD_006)

Accessing support is important to enable people living with dementia to learn about new technologies and avoid disengaging with devices. Therefore, the role of the social network is important to consider when developing new technologies.

Despite these strategies, people living with dementia suggest implementing and learning technologies early because challenges will result in technology becoming unmanageable in the future:

‘I know some people are past the stage of learning, which I will eventually get to. I mean later on it'll be me sat on the other side saying “ohh technology but I can't use it, but I used to be able”’ (PWD_003).

This means that technologies which aim to improve the lives of people living with dementia must be implemented early to give people the best chance at learning and maintaining the skills to use them.

### Theme 2: Motivation to use technology

This theme describes people living with dementia's motivations to use technology which are driven by their own needs, the people around them, and their pre-existing experiences and beliefs about technology. People living with dementia's use of devices suggests they are motivated to use technology as a means of fulfilling their psychosocial needs of personhood: Identity, comfort, attachment, inclusion, and occupation.^43^

**Figure 5.**
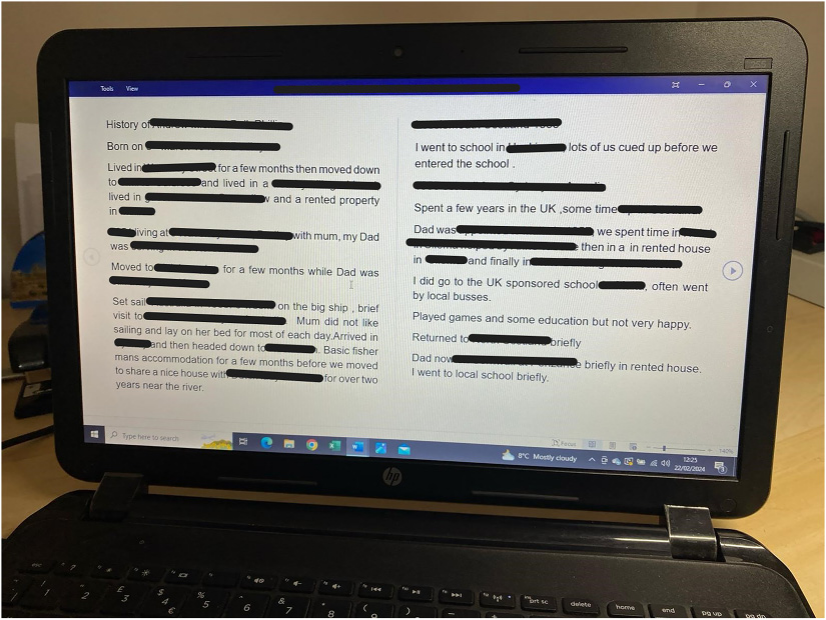
A participant maintaining identity by documenting their history using a laptop computer.

Identity is maintained by using devices to document or connect with individuals’ historical past and current life. Connecting with the historical past is illustrated by individual's using devices to document their life stories and explore their ancestry (see Figure 5):

‘I’ve got things I like myself. The family history I’ve done for years’ (PWD_004).

Connecting with their current life includes sharing their knowledge of dementia with others, such as publicly sharing their dementia story online as highlighted by one participant:

‘I've been on there attending webinars to, to, to speak publicly. I do a lot of that on online. So yeah, they enabled me to tell my story, my dementia story’ (PWD_001).

Comfort is achieved by using technology to facilitate calm and contentment. This is accomplished by using devices to induce relaxing activities such as reading or listening to music. One participant describes creating relaxation videos using their mobile phone:

‘I've taken videos of the sea, you can hear the, the sound of, of the, on the pebbles and yeah. And the sun coming up, which is [pause] Which I don't tire of watching, sometimes it's quite nice' (PWD_005),

Additionally, using technology to monitor health and the environment provides peace of mind leaving people living with dementia feeling safe and content. For example, one participant uses a fit bit to monitor his daily health:

‘One of the other pictures is of the the, the Fitbit. You know, so that's again part of the morning routine to have a look, how am I today? What's my heart rate and that sort of thing? How did I sleep?’ (PWD_009),

And another uses smart doorbells to foster feelings of safety:

‘But, don't wanna panic about things like that. Of course, you imagine you keep thinking about things, but [pause] no, we've got quite a few cameras actually that can, I mean I showed you that one but we’ve got another up in there…’ (PWD_011).

Attachment is fulfilled by using technology to connect with others and the world to reduce isolation. This is achieved by using devices to socialise, such as video calling family who live far away:

‘I use WhatsApp video calls as well to just call my daughters and, and because I can see my grandchildren and they can see me, “hi grandad!” [laughs] “cause they live a long way away”’ (PWD_001)

And by using technology to keep up with current affairs, like one participant who uses a smart speaker for interaction and to gain daily news and information:

‘She says good morning to me and she tells me that what the breaking news is, she tells me what temperature it is and she hopes I have a nice day, which is more conversation than I get out the daughter’ (PWD_006).

**Figure 6.**
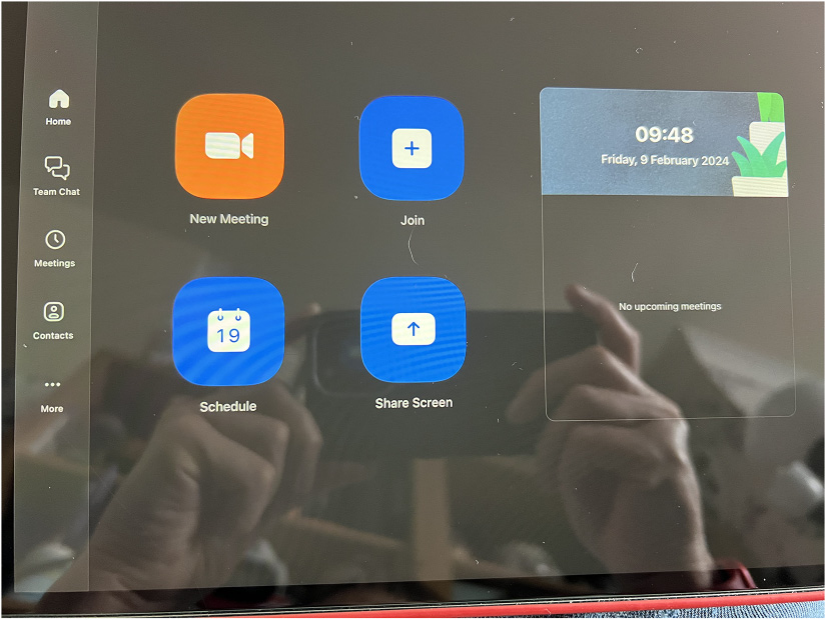
Zoom^©^ used to meet other people with dementia.

Inclusion is reflected in individuals’ involvement in dementia support groups and networks which are facilitated online (see Figure 6). For example, one participant describes connecting internationally with others living with dementia using online videoconferencing:

‘I helped start it up for people with a diagnosis. It was supposed to have been for my area but because of COVID, it didn't mean, location didn't matter, so it meant that we had people from New Zealand, Switzerland, Singapore, joining in on the, meeting. So they, they, they continue now online, once a month’ (PWD_005).

**Figure 7.**
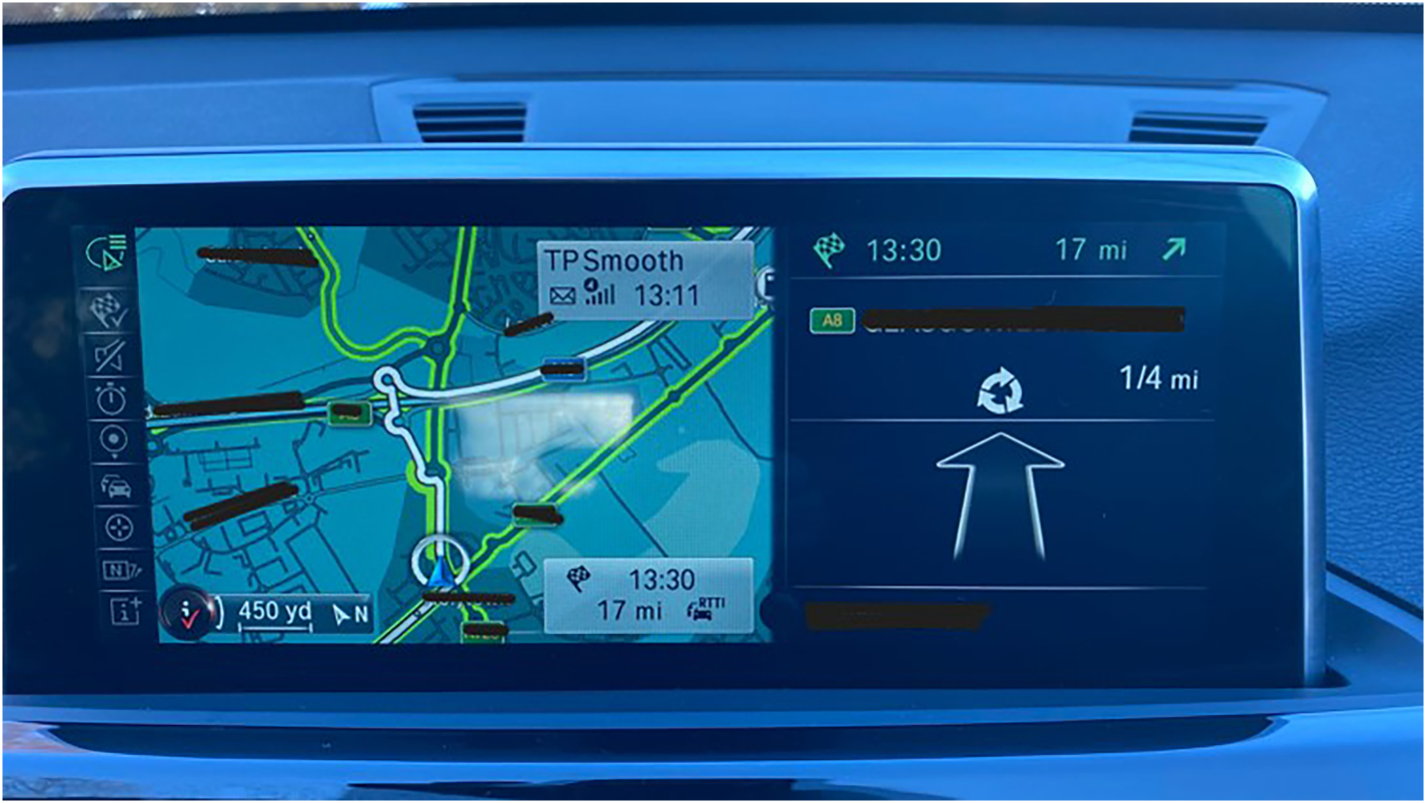
A participant's car sat nav.

Additionally, technology facilitates face-to-face inclusion by supporting people to navigate their physical world using wayfinding apps (see Figure 7). For example, one participant describes his satnav enabling him travel which would not be possible without technology:

‘I feel very vulnerable often now when I'm out and about, never used to, i'm a very confident person, you know travelled all over the world by bus and train, as well as car. But I can't do that now so thank goodness for my sat nav’ (PWD_001).

**Figure 8.**
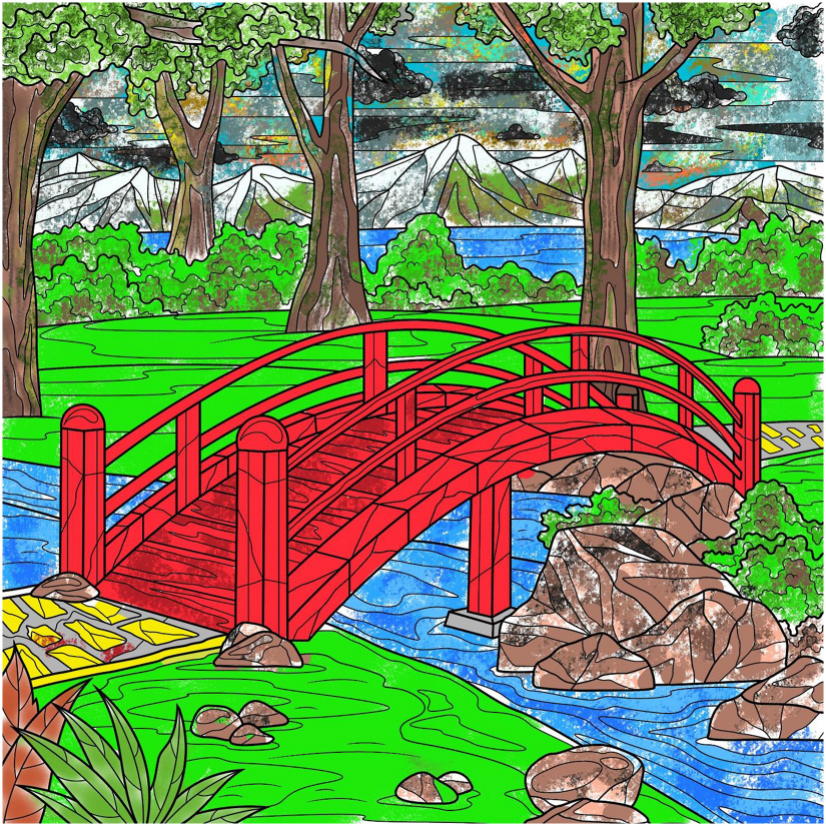
Art created using an electronic tablet.

Occupation is fulfilled by using technology to fill time meaningfully through leisure, or learning. This includes providing opportunities to explore new hobbies, or facilitating hobbies which would not be possible without the support of technology, such as drawing in the case of one participant (see Figure 8):

‘Without it, I would struggle now because with the tremor it becomes a lot harder to, to draw or to try do straight lines or whatever. You'd end up with a squiggly line when you didn't want one’ (PWD_003).

Or learning new skills via online groups as described by another participant:

‘I've also used it with workshops with the group where it's been again like zoom meetings, but we've done things like, had workshops about poetry and all that sort of stuff over zoom, which I've used this laptop for and, art and all other stuff like photography and that sort of stuff’ (PWD_002)

These motivations demonstrate that people living with dementia feel positively about technologies which maintain their personhood and improve their quality of life.

Technology motivation is also driven by family, friends, and individuals outside of the individuals’ usual social networks such as service providers. These individuals provide advice and recommendations for purchasing technology, as highlighted by one participant's decision to explore air fryers:

‘…then someone suggested an air fryer. So I started looking up on YouTube and what have you, and it had pros and cons for different ones, and then the Gadget show did a thing on air fryers’ (PWD_006).

**Figure 9.**
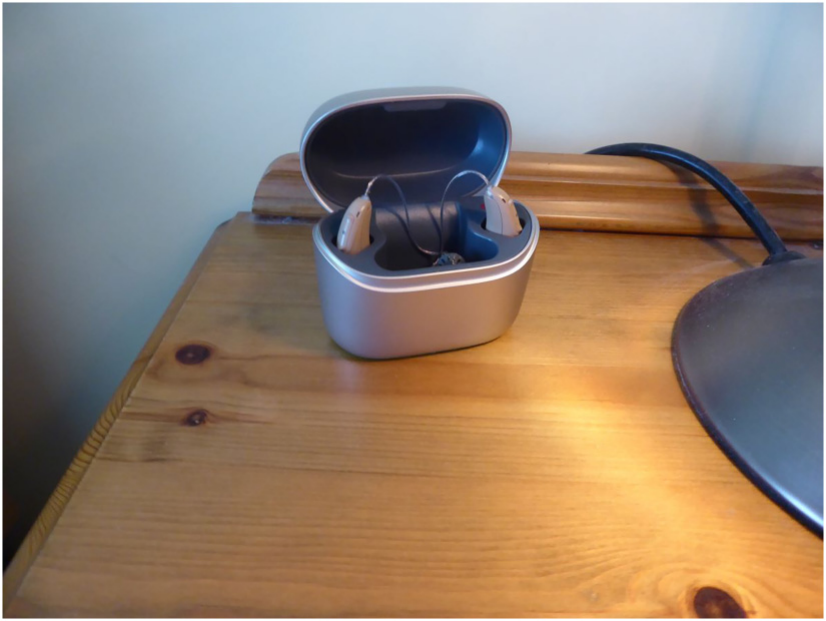
Hearing aids on charge.

Family members may implement the technology without consulting the person living with dementia. This method of implementation also occurs through people gifting devices, or because current devices are outdated and upgraded by the service provider. For example, one participant describes being forced to change her hearing aids because her usual aids were decommissioned by the provider (see Figure 9):

‘…so the old ones you know I could just get them in, get them out they were easy. It would never have occurred to us to get these super-duper ones. But when the NHS changed, changed their, [pause] their producer I suppose [pause]…’ (PWD_010).

Exposure to devices in this way can lead to individuals becoming motivated despite previously be uninterested because their thoughts on its usefulness change. For example, one participant who previously saw no need for an Alexa described finding it useful after her husband initiated it*:*

‘Well at first I didn't think so, you see, I think to me it was just another gadget for him. So yes and, but gradually, he you know, he, he pushed me into doing things. Yeah’ (PWD_004).

Therefore, the needs and preferences of people living with dementia's extended networks such as their family should be considered when implementing devices, as these individuals can be crucial to what technology is purchased.

Finally, people living with dementia have pre-existing feelings and beliefs about technology which have developed throughout their life. Positive experiences in childhood or through work create positive feelings and beliefs which motivate people to use new and future technologies. For example, one participant describes how his father's enthusiasm for photography has influenced his own interest in taking photographs:

‘Mainly because my dad used to be a naval man who always took photos, everything. He was very good [pause] And, and, he said, make sure you can do all these photos, what you're doing, and it er was rather nice thought. Of course, we've still got loads and loads of pictures. I don't know about you, but at least 10,000’ (PWD_011).

Another participant describes using technology frequently in his occupation leading him to be confident about technology in his current life:

‘I've been in IT since I bought, I was in the Royal Air Force at RF St Morgan. In fact in 1980, when I, when I enrolled in the very first RAF Computer literacy course’ (PWD_001).

Such experiences influence cognitive processes by boosting individual's views of themselves as a technology user giving them confidence and motivation to use technologies. Therefore, given that current and future generations are immersed in technology earlier in their lives, digital methods to support later life might become more desirable as skills and confidence are already present.

### Theme 3: Importance of integrating appropriate devices

**Figure 10.**
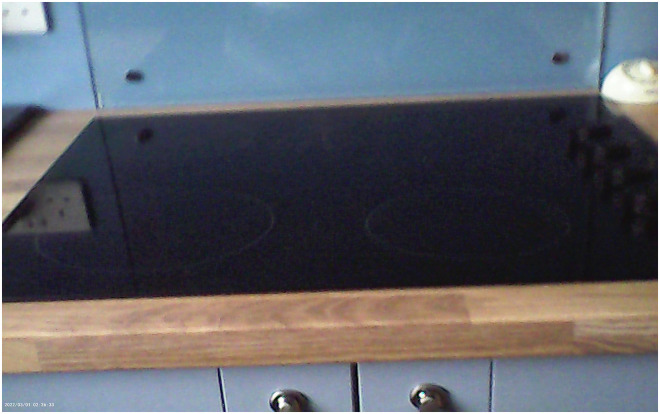
A glass, all black hob which made it difficult for a participant to use.

Positive feelings towards technology are created when devices are appropriate to the needs and preferences of the user, in terms of their design, price, and complexity and are well integrated into the homes and lifestyles. Unsuitable designs, such as primarily black, or small hardware, are challenging due to visual and co-ordination difficulties; this is highlighted by one participant who describes being unable to cook using the hob due to its design (see Figure 10):

‘I used to have to say what, what button is that. Do I need, because like I, it's, it's all little black ones, you know and it's got like a little split in between it and [pause] I used to be able to do some of it, you know it's, but I just couldn't get that into my head. And I, I just used to come say “oh you'll have to do it”’ (PWD_004).

In contrast simple software designs without multiple security measures are more accessible because these rely less on users having to remember passwords which participants describe as stressful:

‘I find difficult to use getting the banking apps. They, they are more stressful to use. More so because of all the security that they got and I can't remember…’ (PWD_003).

Also, designs that demonstrate continuity in layout and format are preferred because this requires less new learning which can be a challenge as dementia progresses:

‘I got fed up with the, you know [pause] they, they have to do everything differently (…) I wanna see an international standard, so people living with dementia, we know where everything is’ (PWD_001).

In terms of complexity, devices that fulfil multiple needs are more desirable to those who have the knowledge and confidence to utilise their many functions. Individuals who lack this prefer simpler devices because they are disheartened when failing to use complex devices. For example, one participant appeared dejected when describing his laptop because he was not using it to its full potential:

‘It's easy for what I use it for . .. As I say I use it for teams [pause] mainly for teams and zoom calls and the Internet. I would probably struggle to use it for, for anything much more than that like, which is a shame because it's quite a nice wee laptop but I would struggle to use it for much more than that’ (PWD_002).

Evidently, a one size fits all approach is insufficient as people living with dementia's needs and preferences for devices are varied. Therefore, consideration as to how devices can be individually tailored or adapted is crucial to consider at the development phase to optimise accessibility.

Willingness to pay for technologies is also important and individual's negotiated price based on cognitive beliefs such as how useful the technology will be. In most cases, usefulness takes precedent over price meaning cheaper alternatives are prioritised:

‘No functionality is more important I think, if I can get the same outcome with something cheaper that doesn't have to be a named brand, then I will do that. But as long as it achieves what I want to achieve’ (PWD_003).

However, in some cases, expensive versions are preferred because these are seen as better quality and better value for money long term:

‘I use the technology so much it's no good going for a cheap solution, cause like fairy liquid you want a good solution. It's going to last longer’ (PWD_009).

Therefore, price should also be an important consideration when developing new dementia technologies because if devices are expensive and not thought to be useful individuals will not purchase them.

Devices must also be incorporated physically into homes to ensure they are used regularly. For some, this involves having technology wired into the home such as smart home heating technology:

‘Yes it is, and of course the, the heating here is all on the floor under floor heating. Well, you know, you say yeah, about your place but we have lots of heat’ (PWD_011).

**Figure 11.**
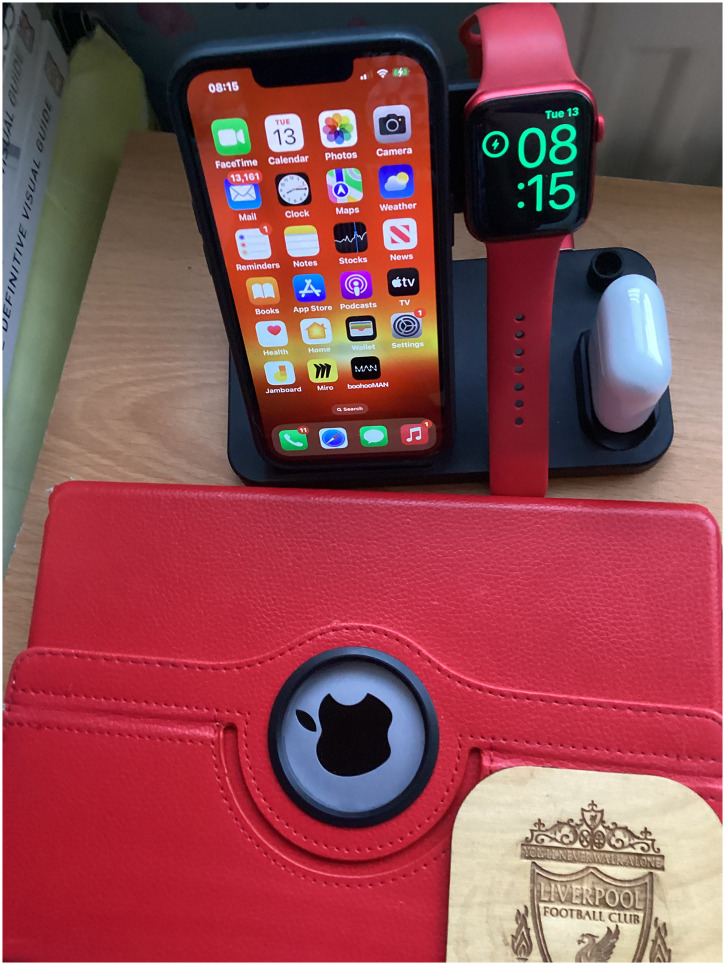
A participant's apple devices which link together at their charging dock.

Others describe devices having a specific place within the home often linked to other devices to create immediate and consistent access (see Figure 11). For example, one participant describes having multiple smart speakers deliberately placed in different locations of her house so she can get support wherever she is:

‘I use that as alarm clock and also if I I'm upstairs like here in the study I can ask questions if I'm sort of question. Downstairs I can also use that, her, as well and for you know different things. But, what I've got there and I'm sure I've put it down is Relish. There's a, that's brilliant’ (PWD_008).

As well as physical location*,* behaviours such as incorporating devices into routines is important, so skills are maintained. When devices break or require updating, relearning is needed which participants describe is a barrier to using the technology:

‘The other thing because there's a gap for delaying me having it on, when it does go on, it has to update everything. So by the time it's updated everything I've gone on to something else’ (PWD_005).

Consistent use and easy access to devices within the home is therefore important because this reduces the need for relearning. This can be achieved by developing devices which can be linked around the home so they can be established into routines.

Successful integration of appropriate devices provides many benefits to people living with dementia in terms of achieving goals and living well. For example, one participant describes how technology enabled him to write a book which was a huge achievement:

‘I’ve wrote a book now I wouldn't have done it because I, at the moment my writing is, is rubbish, so I wouldn't have been able to do it. So it's, it's enabled me to do things that I wouldn't have normally have done’ (PWD_003).

Successful use of technology also allows people living with dementia to avoid feeling emotions which they perceive as undesirable such as anxiety or being a burden. For example, one participant finds wayfinding apps useful because they prevent feelings of vulnerability:

‘It would just be too far out of my comfort zone and I'd be too afraid, not afraid. Yeah, but in a way it's, it's afraid brought about by anxiety, the fear of getting lost, but also the embarrassment of having to be reliant on other people that I don't know whether they're reliable or not’ (PWD_009).

Multiple positive experiences using a device builds confidence and leads to further technology explorations and purchases. For example, one participant describes growing in confidence after being taught how to use a device leading to her independently exploring other features:

‘Oh yeah, it's very much now becoming a two-way street. He taught me [pause] I have dabbled and what have you and expanded and sometimes I have shocked him’ (PWD_006).

This means supporting people living with dementia to have successful technology experiences is crucial because this may be powerful in motivating them to engage in other technologies to support their wellbeing.

### Theme 4: The importance of setting in technology implementation

This theme refers to people living with dementia's negative beliefs about the current societal use of technology, and how technology implementation in settings such as in the high street and workplace has negative consequences for the future and wider society. For some, the gravity of these negative consequences is profound:

‘Both of my girls have you know, of a mind not to have children, right. I feel glad about that because I do believe, what with climate change due to technology and the Victorians and their smoky atmospheres right, and you know doing these great big factories with the bellowing out of the steam engines, which was the technology of the day. What sort of climate are we? You know what sort of planet are we going to have? Like you know what I mean? You're gonna have a robot come. I mean, you've already got them bloody sweeping your bleeding floor for ya’ (PWD_006).

Consequences include paranoia surrounding new technologies such as artificial intelligence which make technology interactions feel unsafe:

‘We have all this AI going on where you think you're talking to a person, but you're not, you're just talking to a robot. (…) they try to put something on screen, so to make you feel you're talking to a person, it's scary because there's nothing like a person (…) but, but you're then gonna have problems that as soon as these androids as soon as they start looking and sounding too human, the human race is going to get absolutely terrified for them’ (PWD_009).

**Figure 12.**
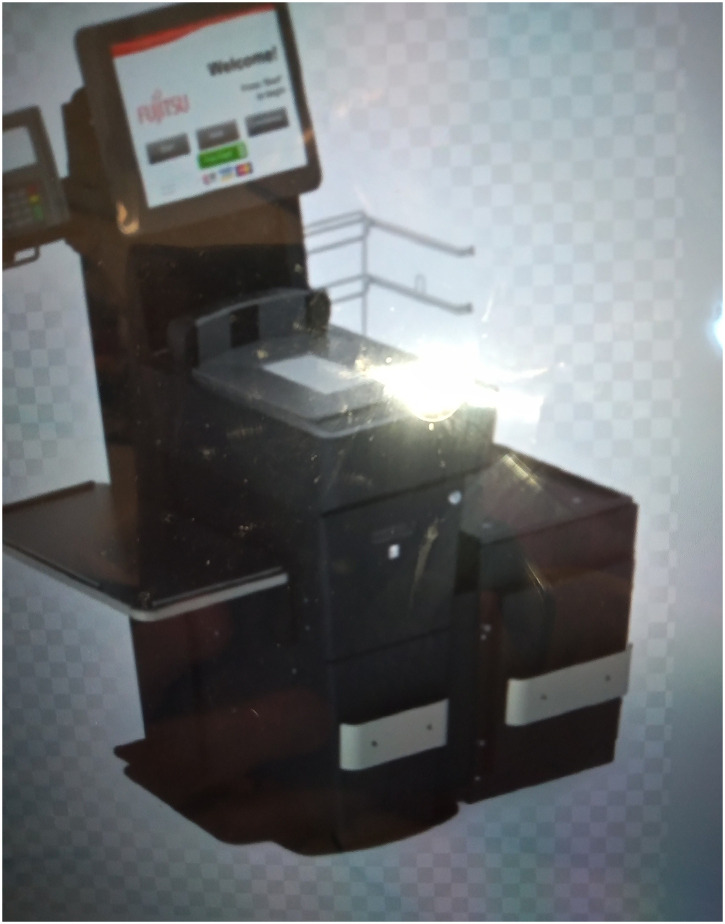
Self-service shopping technologies encountered by a participant.

Other paranoias include scammers, and technology replacing humans leading to face-to-face services being shut down and people losing their jobs (see Figure 12):

‘Asda brought in the self-service till where people who've got a small amount of shopping did it themselves, and they cut her hours. They cut her hours, but not for the same money, Oh no. They cut her hours and cut her wages. And when I met up with her when she told me about this, I took her for a coffee because she hadn't been able to afford to go to a coffee shop and have a coffee for six months’ (PWD_006).

In some instances, these negative perceptions influence behaviour by causing deliberate non-use of technologies outside of the home environment:

‘I had to learn online banking, which is very easy. Yeah, but I, I refused to do it all these years, even though [charity worker] said it's quite easy. I said don't care. I'm not doing it. Because I wanted to put foot fall over the threshold to keep the bank open because I sometimes, I see things like banks being the hub of the community of the High Street. If you start taking banks away, that is the beginning of the end of the High Street’ (PWD_006).

These are important factors to consider when implementing technologies for people living with dementia because those seen to be replacing humans or negatively impacting on existing services may be less accepted than those seen to enhance or supplement.

Despite this, people living with dementia remain hopeful about how technology can be used in the future, if current practices are innovated. Participants were optimistic that future generations of people diagnosed with dementia will benefit more because they will have more technology confidence due to growing up with technology:

‘I think personally that specially for young onset dementia it's, it's actually, could be a really good tool to help provide information, signpost people and be a hub for what's going on locally in your area (…) So I, I think technology could really bring it to the generation that's coming, as well, I think it could be really useful’ (PWD_005).

Therefore, research should continue to explore how technological methods of providing support to people living with dementia can be optimised.

## Discussion

Our findings were interpreted through a lens of empowerment meaning our interpretation of the findings was focused on strength and ability over deficits, thus this research highlights many people living with dementia have access to and use technologies in their everyday lives. Technologies used by people living with dementia rarely included dementia-specific technologies and tend to be off the shelf devices.^15,26,27^ These are often adapted to fulfil assistive technology roles without participants thinking of them as ‘assistive technology’.^15^ Thus, language used when discussing technologies is important, because cognitively, individuals may not identify as using assistive technologies when behaviourally they do.

Liddle^27^ suggests people living with dementia seek out devices appropriate to them in terms of its design, complexity, and price, then employ strategies to help them use technology. This study found similar, with behavioural strategies including getting help from others and tailoring their devices to make them accessible. Some participants initiated these behaviours independently, challenging previous assumptions that interest in technologies must be shared by carers.^15,27,49^ However, all recognised the importance of having support from family, friends, and others to set up and maintain technology skills, especially as dementia symptoms worsened.^27,49–52^ Our explorations of people with dementia's cognitive and affective decision-making indicated that they may be motivated to use technologies which enhance their sense of personhood.^43^ Whilst previous reviews have mapped mobile dementia technologies to this framework,^50^ this study is the first to explore people living with dementia's lived experiences of using everyday technology using Kitwood's^43^ principles. The findings are consistent with previous explorations of technology use in terms of how technology is used. The activities found in this study such as reminiscing and sharing dementia stories, videoconferencing with family and dementia groups, gaming and leisure, wayfinding, and running workshops and self-advocacy events are all present in previous literature.^27,52,53–57^ Interestingly, contrary to previous studies,^27^ our findings also show that cognitively, some people living with dementia are concerned about safety and utilise monitoring technologies such as CCTV type devices to monitor their environment which induces positive feelings of contentment.

Motivation came from social networks and early technology experiences which influenced cognitive and affective processes. Like those described by Wilson and colleagues,^57^ many participants had grown up alongside significant technological advancements and those with significant technological histories through their work or lifestyle expressed more confidence and enthusiasm.^27,57,58^ This also included childhood experiences of technology, where those exposed to technology through their parents’ interests appeared more motivated than those whose parents were adverse or more reluctant. This is useful when considering how technologies might benefit future generations who will already be equipped with the confidence and skills to manage more complex technologies.

Our exploration of cognitive and affective processes also shows people living with dementia have concerns about the implications of technology for wider society including safety and ethical implementation. Cognitively, concerns included technology replacing humans impacting on jobs and services. Whilst this has not previously been explored with people living with dementia, similar concerns have been reported in the healthcare and business sectors.^59–61^ This impacted on behaviour resulting in deliberate non-use of some technologies. Technology non-use can be referred to as a process in which an individual no longer uses a technology after introduction or attempts at use.^27^ Like our findings, non-use could be a conscious choice;^27^ however, unlike other research we found that cognitive beliefs on the morality of technology could cause non-use. This suggests that affective consequences such as moral discomfort may also contribute to the non-use of technology by people living with dementia. Despite this and consistent with previous research,^27,62^ people living with dementia remain hopeful about the benefits technology could bring to others diagnosed with dementia in the future. Thus, technology is likely to be an accepted method to support people living with dementia, if their needs and wants are considered during development.

### Limitations and future directions

Despite efforts to recruit a diverse group of participants, the sample lacked ethnic diversity, as few participants from non-white backgrounds were represented. Although participants were recruited across the whole of England, the geographic location (Norfolk/Suffolk) primarily used for accessing communities is predominantly rural and inhabited by white ethnic groups,^63^ and although provision was made to provide translators for non-native English speakers during interviews, all recruitment resources were written in English creating barriers for non-English speakers. Given that researcher AD who was predominant in the data collection and analysis process is also white the results of the current study are only viewed through a white cultural lens and may be less relevant to other groups. Unfortunately, other studies have had similar challenges meaning that other ethnic groups are globally underrepresented in this area of research.^27,52^ To gather the views of other ethnicities, more consideration should be given to how underserved groups can be better accessed. Also, this study only explored the experiences of 10 people living with dementia. Although this was enough to gain sufficient understandings of the emerging themes about technology experiences within this group, statements about technology use may not be broadly applicable to all people living with dementia

A large portion of recruitment came from the national NIHR database ‘Join Dementia Research’. This meant, researchers were constrained to contact participants using their preferred contact method which was often through a carer. The most common reason for declining invitations to take part in this study came from carer's not believing the study was suitable because their family member could not or did not use technology. This meant, although it was possible to recruit individuals who did or could not use technology, most had some prior interest in technology. Carers as a barrier to recruitment has been identified in other dementia studies. For example, protectiveness and ‘it's not for us’ were dominate reasons why carer's prevented people living with dementia engaging in a psychosocial study despite no direct consultation with the person living with dementia themselves.^64^ Given previous observations that carer's beliefs about what people living with dementia can accomplish with technology don't always align with what is accomplished,^15^ it is crucial that research finds more ways to consult people living with dementia directly to improve their research opportunities in technology research.

## Conclusion

This is a novel approach employing photo-elicitation methods to understand people living with dementia's everyday technology experiences. The findings demonstrate technologies could be a desirable way to provide support to people in the mild to moderate stages of dementia because many of these individuals can use technology with the correct tailoring and support. These findings are useful when considering how technologies might assist future generations who will start their dementia journey already equipped with the confidence and skills to manage more complex technologies.

## Supplemental Material

sj-docx-1-dhj-10.1177_20552076251351538 - Supplemental material for A qualitative exploration of people living with dementia's experiences of using everyday technology Supplemental material, sj-docx-1-dhj-10.1177_20552076251351538 for A qualitative exploration of people living with dementia's experiences of using everyday technology by Annabel Ditton, Shirley Evans, Christopher Fox and Jane Cross in DIGITAL HEALTH
