# Supplementary material for: A qualitative exploration of people living with dementia's experiences of using everyday technology
Source: Digit Health. 2025 Jun 30;11:20552076251351538. doi: 10.1177/20552076251351538 (PMC12217572; doi:10.1177/20552076251351538)
Supplement: sj-docx-1-dhj-10.1177_20552076251351538 - Supplemental material for A qualitative exploration of people living with dementia's experiences of using everyday technology [file sj-docx-1-dhj-10.1177_20552076251351538.docx]

**Supplementary Material 1.** NIHR ‘Join Dementia Research’ Descriptions of Mild and Moderate dementia used to describe dementia severity

Mild Dementia

Everyone experiences symptoms in their own way but the following symptoms may be typical in early or mild dementia:

- Loss of memory for recent events.
- Repeating oneself.
- Becoming slower at grasping new ideas.
- Finding it harder to make decisions.
- Showing signs of confusion.

Moderate Dementia

Everyone experiences symptoms in their own way but the following symptoms may be typical in moderate dementia:

- Need more support and help with day-to-day living.
- May need frequent reminders or help with eating, washing, dressing and to use the toilet.
- Becoming increasingly forgetful and may sometimes repeat the same question or phrase.
- May also fail to recognise familiar people or confuse them with others.
